# MicroRNA-182 improves spinal cord injury in mice by modulating apoptosis and the inflammatory response via IKKβ/NF-κB

**DOI:** 10.1038/s41374-021-00606-5

**Published:** 2021-05-31

**Authors:** Min Fei, Zheng Li, Yuanwu Cao, Chang Jiang, Haodong Lin, Zixian Chen

**Affiliations:** 1 Department of Anesthesiology, Zhongshan Hospital, Fudan University, Shanghai, China; 2 Department of Orthopedics, Zhongshan Hospital, Fudan University, Shanghai, China; 3 Department of Orthopedic Surgery, Shanghai General Hospital, Shanghai Jiaotong University School of Medicine, Shanghai, China

**Keywords:** Spinal cord diseases, Apoptosis, Acute inflammation

## Abstract

Spinal cord injury (SCI) is one common neurological condition which involves primary injury and secondary injury. Neuron inflammation and apoptosis after SCI is the most important pathological process of this disease. Here, we tried to explore the influence and mechanism of miRNAs on the neuron inflammatory response and apoptosis after SCI. First, by re-analysis of Gene Expression Omnibus dataset (accession GSE19890), miR-182 was selected for further study because of its suppressive effects on the inflammatory response in the various types of injuries. Functional experiments demonstrated that miR-182 overexpression promoted functional recovery, reduced histopathological changes, and alleviated spinal cord edema in mice. It was also observed that miR-182 overexpression reduced apoptosis and attenuated the inflammatory response in spinal cord tissue, as evidenced by the reduction of tumor necrosis factor (TNF)-α, interleukin (IL)-6, and IL-1β, and the induction of IL-10. Using a lipopolysaccharide (LPS)-induced SCI model in BV-2 cells, we found that miR-182 was downregulated in the BV-2 cells following LPS stimulation, and upregulation of miR-182 improved LPS-induced cell damage, as reflected by the inhibition of apoptosis and the inflammatory response. IκB kinase β (IKKβ), an upstream target of the NF-κB pathway, was directly targeted by miR-182 and miR-182 suppressed its translation. Further experiments revealed that overexpression of IKKβ reversed the anti-apoptosis and anti-inflammatory effects of miR-182 in LPS stimulated BV-2 cells. Finally, we found that miR-182 overexpression blocked the activation of the NF-κB signaling pathway in vitro and in vivo, as demonstrated by the downregulation of phosphorylated (p‑) IκB-α and nuclear p-p65. Taken together, these data indicate that miR-182 improved SCI-induced secondary injury through inhibiting apoptosis and the inflammatory response by blocking the IKKβ/NF-κB pathway. Our findings suggest that upregulation of miR-182 may be a novel therapeutic target for SCI.

## Abstract (web summary)

overexpression of the microRNA miR-182 promotes functional recovery and reduced histopathological changes in spinal cord injury mice. miR-182 overexpression reduces apoptosis and attenuates the inflammatory response in spinal cord tissues by blocking the IKKβ/NF-κB pathway. These findings suggest that upregulation of miR-182 may be a novel therapeutic target for SCI.

## Introduction

Spinal cord injury (SCI) is a common neurological condition with high mortality and disability worldwide [1]. Although great improvement in treatment methods for SCI has been made, such as drug therapy, surgical treatment, and transplantation therapy, no SCI treatment can achieve a complete cure [2]. A number of studies have revealed that secondary damage is critical in the pathological progression of SCI, with marked effects on neuron apoptosis and the inflammatory response, which can lead to severe neurological damage and dysfunction [3–5]. However, few studies have been conducted on the mechanisms of secondary injury after SCI.

The inflammatory response is a major event in secondary injury in SCI, which is regulated by various pathways including the NF-κB, MAPKs, and IL-6R/JAK-STAT pathways, though the NF-κB pathway has been the most thoroughly studied [6–8]. The degradation of IκB mediates activation of NF‐κB signaling pathway, and the IκB kinase (IKK) complex can promote the phosphorylation of IκB [9]. In the IKK complex, the catalytic subunit IKKβ has an important role in the phosphorylation of the IκB protein [10]. In spinal cord tissue, NF-κB is usually activated in nerve cells and microglia after SCI. Once activated, the secretions of a large number of inflammatory cytokines such as IL-1, TNF-α, IL-6, aggravated secondary damage after SCI [11, 12]. Therefore, suppression of the NF‐κB pathway limits pro‐inflammatory phenotypes and improves the secondary injury.

MicroRNAs (miRNAs) are single-stranded non-coding RNAs that bind to the target mRNAs and interfere their translation [13, 14]. Recent evidence indicates that miRNAs are abnormally expressed in plasma of SCI patients, which suggests that miRNAs can function as novel biomarkers for disease diagnostics [15, 16]. As well as global miRNA changes, the roles of specialized miRNAs in SCI have been elucidated through the use of animal models [17, 18]. For example, Wang et al. showed that miR-940 was decreased in the spinal cords of mice with SCI, and overexpression of miR-940 promoted the recovery of SCI by inhibiting inflammation [19]. Xu et al. showed that miR-124 improved functional recovery through suppressing neuronal cell apoptosis in SCI rats [20]. However, limited studies have been published on the role of miRNAs in the pathophysiology of secondary damage after SCI.

In the present study, through the re-analysis of the Gene Expression Omnibus (GEO) dataset (accession GSE19890), miR-182 was identified as one of the downregulated miRNAs with the smallest change in a mouse model of contusive SCI. Subsequently, the function of miR-182 was further elucidated using agomir-miR-182 injection in this model. Moreover, the underlying mechanisms of the miR-182-mediated protective role in SCI were investigated in vitro and in vivo. Our findings may increase our understanding of miR-182 regulation after SCI and promote the development of therapeutic measures for SCI.

## Materials and methods

### Animals and grouping

Female C57BL/6 mice (12 weeks, 18–24 g) were purchased from SLAC Laboratory Animal Co., Ltd (Shanghai, China) and housed under standard conditions (12 h light-dark cycle, 25–27 °C, ~40% humidity) with free access to food and water throughout the duration of the experiments. All experimental procedures were approved by the Animal Ethics Committee of Zhongshan Hospital, Fudan University. The animals were randomly divided into four groups: Sham (*n* = 36), SCI (*n* = 36), SCI + agomir-miR-182 (*n* = 36), and SCI + agomir-negative control (NC) (*n* = 36). All groups were subdivided into six time points: 1-, 3-, 7-, 14-, 21- and 28-day post-SCI (*n* = 6 per time point). All animals survived the experimental period without adverse effects and were included in the data analysis.

### SCI model establishment

Mice were anesthetized with pentobarbital sodium (50 mg/kg) by intraperitoneal injection, and a laminectomy was performed at thoracic vertebra level T10 as previously described [21], and the spinal cord was exposed. SCI was induced by the weight drop (10 g) from a 2.5-cm height on a T10 spinal cord. Sham-injured animals were only subjected to a laminectomy without hitting the T10 spinal cord. Animal procedures were approved by the Institutional Animal Care and Use Committee of Zhongshan Hospital, Fudan University.

### Injection of agomir-miR-182

Mice were randomly divided into four groups to determine the role of agomir-miR-182: SCI group, Sham group, SCI + agomir-miR-182 group, and SCI + agomir-NC group. In the SCI + agomir-miR-182 group/agomir-NC group (*n* = 6/group/time), the mice were subjected to SCI and then the mixed miR-182 agomir (20 µM, 2 µL) was injected into the mice using a glass micropipette (tipdiameter 20–40 µm) via intrathecal injection. The injection rate was 0.2 μL/min, and the needle was left in place for an additional 5 min before being slowly withdrawn. The dose of the agomir-miR-182 was based on the results obtained from in spared nerve injury (SNI) model [22]. After 4 weeks, all mice were sacrificed following pentobarbital (50 mg/kg, i.p) anesthetization, and subsequently, a 10 mm long segment of the spinal cord centered at the injury epicenter was harvested for further experiments. The miR-182 agomir (5′-UUUGGCAAUGGUAGAACYCACACCG-3′) and its scrambled negative control (5′-UUCUCCGAACGUGUCACGUTT-3′) were synthesized by GenePharma (Shanghai, China).

### Basso, Beattie, and Bresnahan (BBB) score

BBB score was performed to test locomotor function of mice at 1, 3, 7, 14, 21, and 28 days post injury in an open field, and was scored between 0 (no observable hind-limb movements) and 21 (normal locomotion) as described previously [23]. At each time point, the outcome value for each mouse was obtained by averaging motor score from the observation and scoring of behaviors involving the hindlimbs. The scores were recorded by two well-trained investigators who were blind to the experiments.

### HE and Cresyl violet staining

A 10 mm long segment of the spinal cord containing the epicenter from indicated groups were fixed with 4% paraformaldehyde (Solarbio, China), embedded in paraffin, and cut into 10-μm thick serial sections. One set of tissue sections were used for routine HE staining (Beyotime, China). The others were put onto Superfrost Plus Slides and every 40th section was stained with 0.5% cresyl-violet acetate and imaged using BX51 light microscope (Olympus Inc., Tokyo, Japan). Using Image-Pro Plus 6.0 (Media Cybernetics, USA) software, the lesion area and spared tissue area were outlined and quantified as described previously [24].

### Spinal cord water content measurement

The spinal cord tissues obtained in the above experimental procedure at 7 days post injury were immediately weighted, and then the dry weight of spinal cord tissues was obtain at 100 °C for 24 h. The ratio of wet-to-dry weight calculated as follows: [(wet weight − dry weight)/wet weight] × 100%.

### TUNEL staining

Apoptosis in spinal cord tissues was detected by Situ Cell Death Detection Kit (cat no.11684817910, Roche). The 10 mm spinal cord sections were deparaffinized and rehydrated in an alcohol gradient. After washing with PBS, TUNEL reaction mixture preparation and staining were performed according to the manufacturer’s protocol. The images were captured by the inverted fluorescence microscope (×400 magnification, Olympus Inc., Tokyo, Japan). The TUNEL-positive cells in 10 random fields of each section were counted for analysis.

### Immunohistochemical (IHC) and immunofluorescent analysis (IFA)

IHC and IFA staining was performed as previously described [25], with specific antibody for caspase-3 (cat no. 9662), p-IκBα (Ser32 cat. no. 2859), and nuclear p-p65 (cat. no. 3033). All antibodies were obtained from Cell Signaling Technology, Inc., Danvers, MA, USA. Images of IHC were photographed using an Olympus BX51 light microscope (Olympus Inc., Tokyo, Japan) and the images of IFA were captured by fluorescence microscopy (Olympus Inc., Tokyo, Japan) at ×200 magnification. Positively stained cells of IHC were observed and counted by the Image-Pro Plus image analysis management system (Media Cybernetics, Rockville, MD). Five random fields from each slide were selected for capture and counting of positive cells.

### ELISA assay

Spinal cord tissue was dissected and placed in pre-cooled PBS buffer, and then homogenized with a homogenizer. The supernatant was collected by centrifugation at 3000 r/min for 20 min at 4 °C. For cultured cells, the supernatant was collected after treatment, and the supernatant was carefully collected by centrifugation at 2000 r/min for 20 min. IFN-α (cat no.BMS6027), IL-1β (cat no.BMS6002), IL-6 (cat no. BMS603-2), and IL-10 (cat no. 88-7105-22) were measured by ELISA kit (all from Thermo Fisher Scientific, Inc., Waltham, MA, USA) according to manufactures.

### miRNA microarray

Microarray dataset GSE19890 was obtained from the National Center for Biotechnology Information (NCBI) GEO database (http://www.ncbi.nlm.nih.gov/geo) to identify SCI associated miRNAs. GSE19890 dataset was based on miRCURY LNA microRNA Array, v.11.0 platform. GEO2R (www.ncbi.nlm.nih.gov/geo/geo2r/), an interactive web tool was applied to compare the samples in two different groups under the same experimental condition. |log_2_(Fold Change)| (|log_2_(FC)|) was selected as the criteria to screen the statistically significant differentially expressed-miRNAs (DE-miRNAs) [26]. The procedure and imaging processes were as described previously [27].

### RNA isolation and quantitative RT-PCR

Total RNA was extracted from spinal cord and cells with a miRNeasy Mini kit (Qiagen GmbH, Hilden, Germany). Reverse transcription of miR-182 was synthesized using the miScript II RT kit (Invitrogen, Carlsbad, CA). miR-182 expression was measured using the Exiqon SYBR Green Master Mix (Exiqon, Vedbaek, Denmark) on an ABI Prism 7900 HT (Applied Biosystems). The primers used for were as follows: miR-182 RT primer 5′-GTCGTATCCAGTGCAGGGTCCGAGGTATTCGCACTGGATACGACAGCCTACGGTGT-3′; forward: 5′-CGTCCTTTGGCAATGGTAGAACTC-3′, reverse: 5′-GCAGGGTCCGAGGTATTC-3′; U6 forward: 5′-GCTTCGGCAGCACATATACTAAAAT-3′, reverse: 5′-CGCTTCAGAATTTGCGTGTCAT-3′. The relative expression of each gene was calculated using the 2^−∆∆Ct^ method [28].

### Cell culture and treatment

BV-2 cells were widely used to induce cell injuries to mimic the in vitro model of SCI [29]. The BV-2 cell line was obtained from ATCC (Manassas, VA, USA) and maintained in DMEM/F12 (Gibco; Thermo Fisher Scientific, Inc., Waltham, MA, USA) containing 10% FBS (Gibco), and 1% penicillin and streptomycin (Sigma-Aldrich, St. Louis, MO, USA) in 5% CO_2_ at 37 °C.

For the induction of inflammation and apoptosis in the cultured BV-2 cells, 100 ng/ml of lipopolysaccharide (LPS) were used to stimulate cells (Sigma-Aldrich, St Lousis, MO, USA) for 4 h at 37 °C as previously reported [30].

### Cell transfection

When BV-2 cells grown to about 80% confluence in six-well plate, 20 nM agomiR-182 or 2 μg pcDNA-IKKβ were transfected into cells at 37 °C for 48 h, using Lipofectamine^®^ 2000 (Invitrogen). The agomir-miR-182 and agomiR-negative control (NC), were obtained from RiBoBio (Guangzhou, China). IKKβ overexpressing vector pcDNA-IKKβ and pcDNA vector were constructed by Qiagen (USA).

### Caspase-3 activity and NF-κB activity assay

After treatment, total protein was extracted using RIPA buffer (cat no.P0013B, Beyotime Institute of Biotechnology) and protein concentration was evaluated using the bicinchoninic acid assay (cat no.P0010S, Beyotime Institute of Biotechnology), according to the manufacturer’s protocols. Then, the activity of caspase-3 was evaluated using a caspase-3 activity kit (cat no.C1115, Beyotime Institute of Biotechnology) according to the manufacturer’s protocols.

The NF-κB activity was assessed as previously described [31]. Briefly, BV-2 cells were plated in six-well plates at a concentration of 5 × 10^4^ cells/well for 24 h, and then transfected with 2.5 µg of a NF-κB reporter luciferase construct. After 6 h, the cells were washed and then co-transfected with agomiR-182 and pcDNA-IKKβ for 24 h. The cells were then washed in PBS and harvested in 500 µl 1× passive lysis buffer. Promega luciferase assay kit was performed to quantify the Luciferase activity using a on a luminometer.

### Luciferase reporter assay

pGL3-IKKβ wide type (Wt) or pGL3-IKKβ mutant type (mut) plasmids were co-transfected with 20 nM agromiR-182 into BV-2 cells in 24-well plates (2 × 10^5^/well) using Lipofectamine 2000 (Invitrogen). At 24 h post-transfection, the double luciferase activities were analyzed using the Dual-Luciferase Reporter Assay system (Promega Corporation) and normalized to Renilla luciferase activity.

### Western blot analysis

Western blot was performed as previously described [29]. Briefly, 40 μg extracted protein samples from spinal cord and cells were transferred onto a PVDF (Millipore) membrane and then this membrane was blocked with 5% skim milk for 2 h at room temperature, followed by incubation with primary antibodies against cleaved-caspase-3 (cat. no. 9664), p-IκBα (Ser32 cat. no. 2859), IKKβ (cat. no. 9188), IκBα (cat. no. 4812), nuclear p-p65 (cat. no. 3033), p65 (cat. no. 8242), IL-6 (cat. no. #12912), IL-10 (cat. no. 12163), IL-1β (cat. no. 12703), TNF-α (cat. no. 11948), Bax (cat. no. 14796), Bcl-2 (cat. no. 4223), cleaved PARP (cat. no. 5625) and β-actin (cat. no. #4970) at 4 °C overnight. All antibodies were obtained from Cell Signaling Technology, Inc and the dilution was 1:1000. Subsequently, the blots were incubated with appropriate secondary antibodies (cat. no. 7074; Cell Signaling Technology, Inc. 1:2000) for 1 h at room temperature. The protein bands were developed using ECL kit (GE Healthcare) and blot bands were quantified with ImageJ version 1.46 (Rawak Software, Inc. Munich, Germany).

### Statistical analysis

Statistical analysis was performed using GraphPad Prism (version 5.0, Inc., La Jolla, CA, USA). Data were recorded as means ± SD. Differences among multiple groups were analyzed by one-way analysis of variance with Tukey’s post hoc test, and differences between two groups were analyzed by Student’s *t* test. A *p* value < 0.05 was considered significant. Each experiment was repeated at least three times.

## Results

### miR-182 was downregulated in spinal cords of SCI mice

**Fig. 1:**
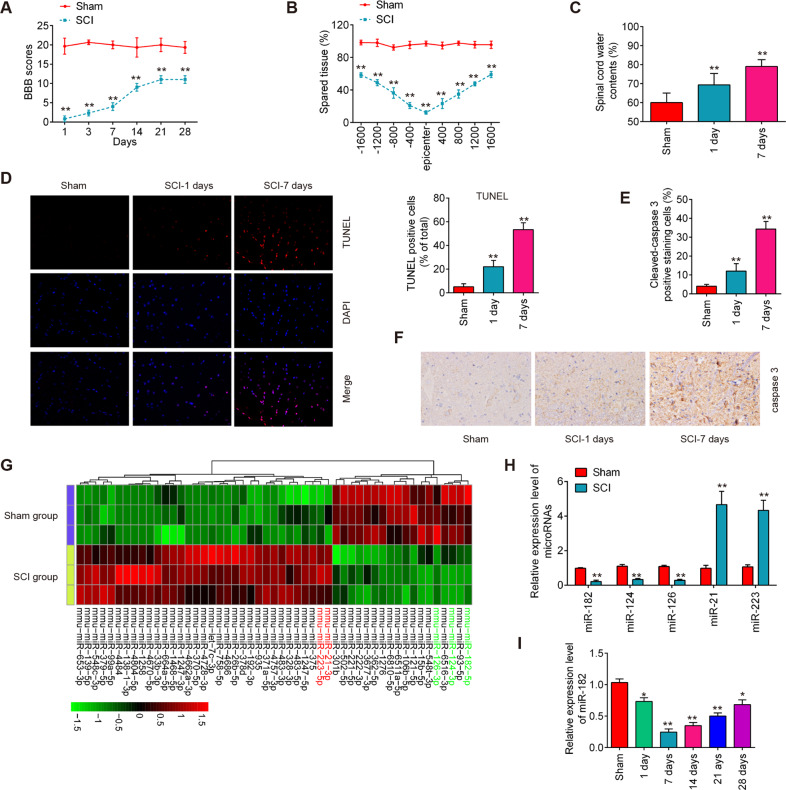
miR-182 was downregulated in the spinal cords of SCI mice. **A** The BBB scores at 1, 3, 7, 14, 21, and 28 days after SCI were shown for all groups of mice (*n* = 6/group). **B** Quantification of spared tissue within the injury site, and 1600 μm rostral and caudal to the epicenter, 28 days post injury (*n* = 6/group). **C** Spinal cord water content was assessed using wet-to-dry weight method (*n* = 6/group). **D** Apoptosis was determined by TUNEL staining at 1 and 7 days post injury (*n* = 6/group). **E**, **F** The expression of casapse-3 was measured by IHC at 1 and 7 days after SCI (×200 magnification) (*n* = 6/group). **G** A cluster heap map was used to present the upregulated and downregulated miRNAs from GSE19890. **H** qRT-PCR was performed to detect the expression of miR-182, miR-124, miR-126, miR-21, and miR-223 (*n* = 6/group). **I** qRT-PCR was performed to determine the expression levels of miR-182 in spinal cord tissues from mice at 1, 7, 14, 21, and 28 days after SCI (*n* = 6/group/time). Data represent the mean ± SD of three independent experiments. *p < 0.05, **p < 0.01 vs. Sham group.

We established an experimental model of SCI mice injury in vivo as previously described [32], and then evaluated the behavioral analyses using BBB motor rating scale. As shown in Fig. 1A, mice from sham group showed no locomotor impairment and maintained full marks in the BBB score for 28 days. Immediately after SCI, mice in SCI group showed paralysis of both hindlimbs. Although motor functions gradually improved until the end of the experiment, much lower BBB scores were observed in SCI group compared with the Sham group. Cresyl violet staining showed that mice in SCI group showed a significant decrease in the amount of spared tissue in the rostral and caudal directions from the injury epicenter (Fig. 1B). It was also observed that the water contents in spinal tissue samples were time-dependently increased in SCI group compared with Sham group (Fig. 1C). In addition, compared with the sham group, a greater number of TUNEL-positive cells were observed in the SCI group, as determined by TUNEL staining assay (Fig. 1D). Concomitantly, the expression of caspase-3 in spinal tissues, an established indicator of apoptosis [33], were significantly increased at 1 d and 7 d post injury (Fig. 1E, F), as determined by IHC. All data indicted SCI model was successfully constructed, and SCI can induce neuronal apoptosis.

In order to identify miRNAs associated with SCI, microarray dataset GSE19890 was retrieved from GEO database. Various miRNAs showed differential expression in the microarray analysis, including 33 upregulated miRNAs and 18 downregulated miRNAs compared with Sham group (Fig. 1G). To further verify these differential expression of miRNAs, the 5 miRNAs (3 most significantly upregulated or 2 downregulated miRNAs) were selected and quantified by qRT-PCR analysis. It was shown that miR-182, miR-21, and miR-223 were significantly increased, while miR-124 and miR-126 were markedly decreased in the spinal cord tissues (Fig. 1H), which are consistent with previous reports [20, 24, 34, 35], indicating the reliability of this microarray. But, in the present study, miR-182 exhibited the most downregulated changes in spinal tissues of SCI mice, and the anti-inflammatory effects of miR-182 have been identified in several types of cells [36–40]. Additionally, two previous studies have reported that miR-182 improved the neurological function of rats in SNI and SCI models [22, 41]. However, whether miR-182 can exert protective effects against the inflammatory response and apoptosis in the secondary injury of SCI remains unclear. Therefore, we focused on miR-182 for further investigation.

Next, the expression change of miR-182 was calculated in spinal cord tissues of mice at different time points. As shown in Fig. 1I, the miR-182 expression levels were reduced at different time points after SCI compared to the expression in the sham group. The decrease in level of expression of miR-182 was minimal at 7 days post injury and then its expression was gradually increased persisted until 28 days after injury. All data indicates that miR-182 may be involved in the pathogenesis of SCI.

### Agomir-miR-182 improved functional recovery and suppressed neuron apoptosis

**Fig. 2:**
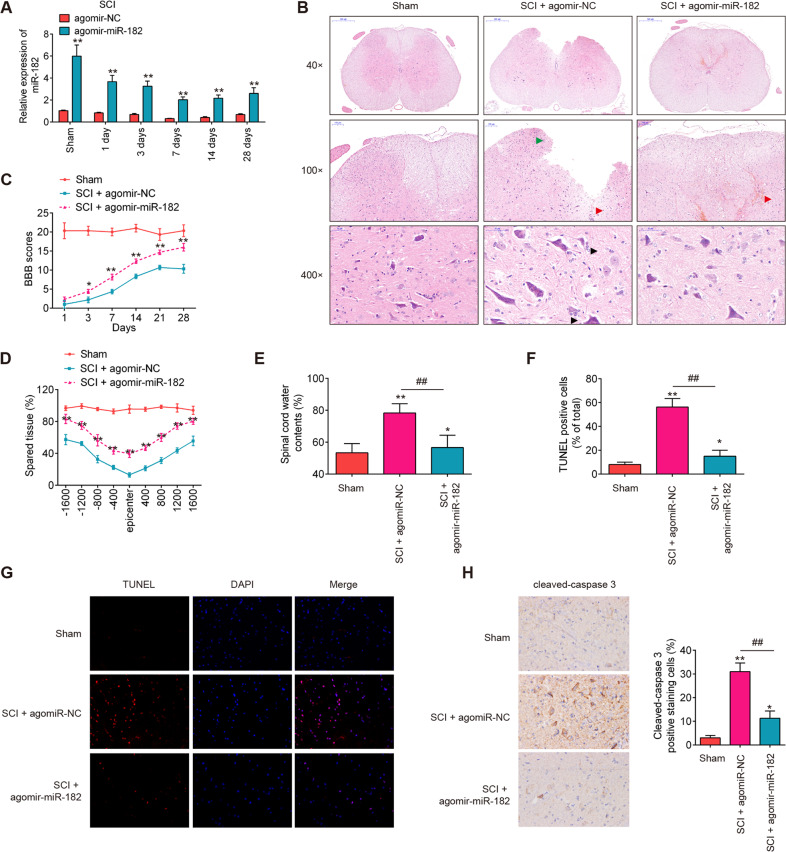
Agomir-miR-182 improves recovery of SCI mice by reducing apoptosis. The mice were subjected to SCI and treated intrathecally with agomir-miR-182/agomir-NC (2 μl, 20 µM). At indicated time, the animals were sacrificed following pentobarbital sodium (50 mg/kg, i.p) anesthesia, and subsequently, a 10 mm long segment of the spinal cord was harvested for further experiments. **A** qRT-PCR was performed to determine the expression levels of miR-182 in spinal cord tissues at 1, 3, 7, 14, 21, and 28 days after agomir-182 injection (*n* = 6/group/time). **B** The staining images of spinal cord tissues following Hematoxylin and eosin (HE) staining (*n* = 6/group). The red blood cells is indicated with red arrow. The inflammatory cell infiltration is indicated with green arrow. The glial cells proliferation and satellitosis (microglial cells surrounding neurons with swollen and prenecrotic neurons) is indicated with black arrow. **C** The BBB scores at 1, 3, 7, 14, 21, and 28 days after SCI were shown for all groups of rats (*n* = 6/group/time). **D** Quantification of spared tissue within the injury site, and 1600 μm rostral and caudal to the epicenter, 7 days post injury (*n* = 6/group). **E** Spinal cord water content was assessed using wet-to-dry weight method (*n* = 6/group). **F**, **G** TUNEL staining of neuronal apoptosis at 1 and 7 days post injury (*n* = 6/group/time). **H** The expression of casapse-3 was detected by IHC at 1 and 7 days post injury (*n* = 6/group/time). Data represent the mean ± SD of three independent experiments. *p < 0.05, **p < 0.01 vs. Sham group; ^##^*p* < 0.01 vs. SCI + agomir-NC group.

To further examine the impact of miR-182 in behavioral motor functions after SCI, agomir-miR-182 and agomiR-NC were intrathecally injected into SCI mice. The agomir-miR-182 transfection efficiency was assessed by qRT-PCR. As shown in Fig. 2A, miR-182 was significantly increased in spinal cord tissues of SCI mice from 1 to 28 d post injury, compared with agomiR-NC group. Histopathologic changes were assessed using HE staining. As shown in Fig. 2B, the sham group displayed a well-defined border between gray matter and white matter, and neurons were abundant. In the injury group, tissue sections contained numerous red blood cells along the injury site with abundant inflammatory cells, particularly glial cells proliferation and satellitosis (microglial cells surrounding neurons with swollen and prenecrotic neurons). Moreover, the injury group had a disordered spinal cord structure including indiscriminate structures between gray matter and white matter, the number of neurons was lowered considerably. However, treatment with agomir-miR-182 attenuated injury remarkably. The structure of the spinal cord was better and the number of neurons greater than that in the injury group (Fig. 2B) The BBB score was performed to evaluate hindlimb motor function recovery of SCI mice following agomir-miR-182 injection. As shown in Fig. 2C, agomir-miR-182 injection significantly improved BBB scores for up to 4 weeks compared with the SCI plus agomir-NC group. Meanwhile, we found that the amount of spared tissue was markedly increased in SCI + agomir-miR-182 group compared with the SCI plus agomir-NC group, indicating that agomir-miR-182 can reduce lesion size in SCI mice (Fig. 2D). In addition, the spinal cord wet-to-dry ratio was evaluated to indicate the spinal cord water edema and we found that agomir-miR-182 treatment dramatically decreased the spinal cord water edema, compared with the SCI plus agomir-NC group (Fig. 2E). Furthermore, the effect of miR-182 on apoptosis in the SCI mice was analyzed by TUNEL staining. It was found that agomir-miR-182 injection resulted in a marked reduction of TUNEL-positive cells, compared with the SCI plus agomir-NC group (Fig. 2F, G). Finally, the expression of caspase-3 in spinal cord tissues was notably suppressed by agomir-miR-182, as determined by immunohistochemistry (IHC) staining (Fig. 2H). Taken together, miR-182 upregulation could promote functional recovery and protect neurons apoptosis in SCI mice.

### Agomir-miR-182 injection inhibited the inflammatory response in SCI mice

**Fig. 3:**
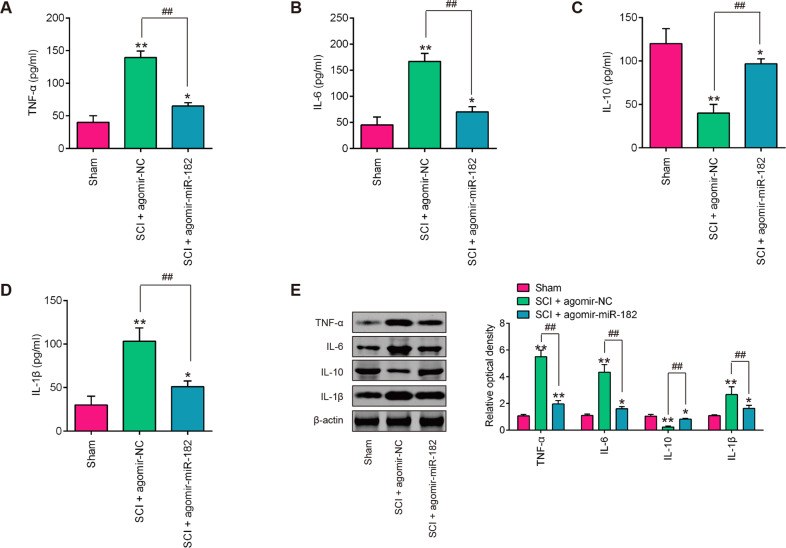
miR-182 inhibited the inflammatory response in SCI mice. The mice were subjected to SCI and treated intrathecally with agomir-miR-182/agomir-NC (2 μl, 20 µM). At 7 days post injury, the animals were sacrificed following pentobarbital (0.3 g/kg, i.p) anesthesia, and subsequently, the spinal cord tissues were harvested for further experiments. **A**–**D** ELISA analysis of TNF-α, IL-6, IL-10, and IL-1β in serum of SCI rat after agomir-miR-182 injection (*n* = 6/group). **E** Western blot analysis of TNF-α, IL-6, IL-10, and IL-1β in spinal cord tissues (*n* = 6/group). Data represent the mean ± SD of three independent experiments. *p < 0.05, **p < 0.01 vs. Sham group; ^##^*p* < 0.01 vs. SCI + agomir-NC group.

To further evaluate the influence of antagomiR-182 on the inflammatory response, the releases of TNF-α, IL-6, IL-1β, and IL-10 in serums of SCI mice were measured by ELISA. As shown in Fig. 3A–D, compared with the sham group, the expression levels of TNF-α, IL-6, IL-1β were significantly increased, but IL-10 was markedly decreased in SCI mice. However, agomir-miR-182 treatment reduced the expression of these pro-inflammatory cytokines, while enhanced the levels of IL-10 induced by SCI. The suppressive effects of agomir-miR-182 on SCI-induced inflammatory response were also confirmed by western blot analysis in spinal tissues (Fig. 3E). These data suggest that agomir-miR-182 suppressed the SCI-induced inflammatory response in mice.

### Overexpression of miR-182 suppressed the inflammatory response and apoptosis in an SCI cell culture model

**Fig. 4:**
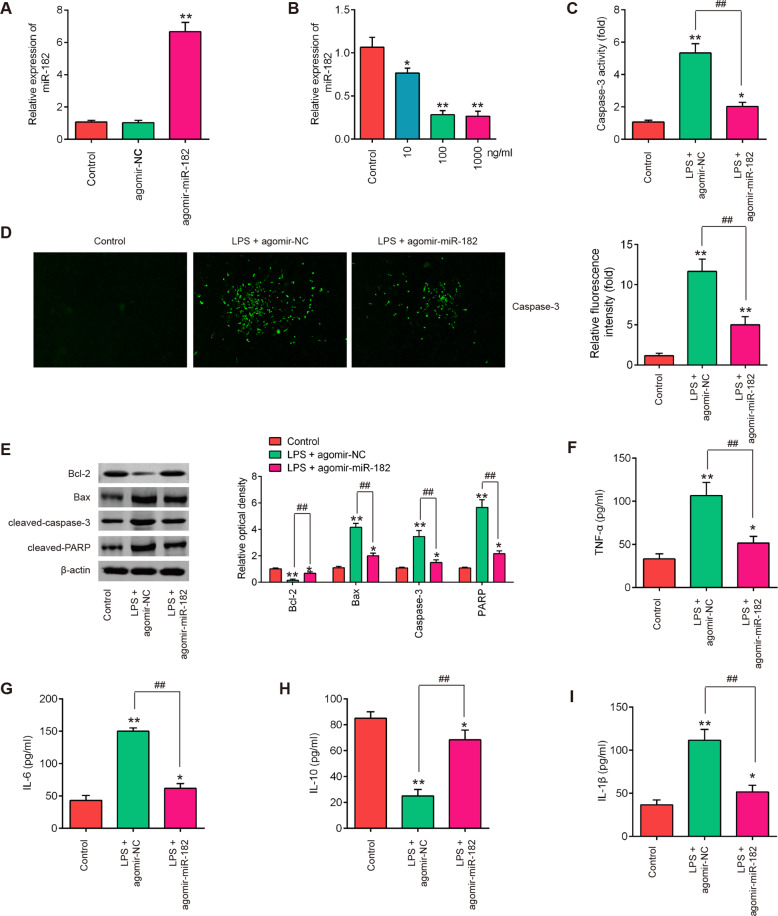
Overexpression of miR-182 suppressed the inflammatory response and apoptosis in an SCI cell culture model. **A** Agomir-miR-182 was added to the cultured BV-2 cells (1 × 10^6^/well) and incubated for 24 h, and then the transfected efficiency of miR-182 was detected by qRT-PCR analysis. **B** BV-2 cells (1 × 10^6^/well) were treated with different concentrations of LPS (10, 100, and 1000 ng/ml) for 24 h, and the expression of miR-182 was detected by qRT-PCR analysis. Agomir-miR-182 was added to the cultured BV-2 cells (1 × 10^6^/well) 4 h prior to LPS treatment and incubated for 24 h, and then cells were harvested for subsequent experiments. **C** Activity of caspase-3 was measured using a commercial kit. **D** The protein expression level of caspase-3 was detected by IFA. **E** The protein expression levels of Bcl-2, Bax, cleaved-caspase-3, and cleaved-PARP were detected by western blot analysis. **F**–**I** The expression of TNF-α, IL-6, IL-10, and IL-1β were measured by ELISA analysis. Data represent the mean ± SD of three independent experiments. *p < 0.05, **p < 0.01 vs. Control group; ^##^*p* < 0.01 vs. LPS + agomir-NC group.

As already known, LPS-induced BV-2 cell injury model has been widely used to mimic the pathological characteristics of SCI in vitro [42]. To further explore the functions of miR-182 in LPS-induced BV-2 cell injury, the agomir-miR-182 was added to BV-2 cells 4 h prior to LPS treatment. qRT-PCR assay showed that miR-182 was significantly increased after agomir-miR-182 transfection in BV-2 cells (Fig. 4A). Following treatment of the BV-2 cells with various concentrations of LPS (10–1000 ng/ml), miR-182 was downregulated in LPS-treated BV-2 cell, and this effect was dose-dependent (Fig. 4B). Thus, 100 ng/ml LPS was selected as the appropriate concentration in the subsequent experiments, which is consistent with a previous study [43]. Then, we evaluated whether miR-182 affects apoptosis in this SCI cell culture model. The data showed that compared with control group, the activity of caspase 3 in LPS plus agomir-NC group was concomitantly upregulated, and the increase was significantly reduced in the presence of agomir-miR-182 (Fig. 4C). The expression of caspase-3, determined as using IFA assay, was found to be increased in response to LPS stimulation, whereas agomir-miR-182 treatment significantly inhibited the LPS-induced caspase-3 expression (Fig. 4D). In addition, the expression of Bcl-2 in LPS plus agomir-NC group was significantly decreased, and the expression of Bax, cleaved caspase-3 and cleaved-PARP were obviously increased, compared with control group. As expected, these effects of LPS on apoptotic related protein expression were attenuated after agomir-miR-182 treatment, suggesting that miR-182 upregulation suppressed LPS-induced apoptosis in BV-2 cells (Fig. 4E). The impact of miR-182 overexpression on the secretions of inflammatory cytokines was further assayed. As shown in Fig. 4F-I, agomir-miR-182 treatment markedly inhibited the expression levels of pro-inflammatory cytokines (TNF-α, IL-6, IL-1β), but promoted the expression of IL-10 in LPS plus agomir-miR-182, compared with LPS plus agomir-NC group. The data suggest that miR-182 upregulation protected the BV-2 cells from LPS-induced apoptosis and the inflammatory response.

### IKKβ is a functional target of miR-182 in BV-2 cells

**Fig. 5:**
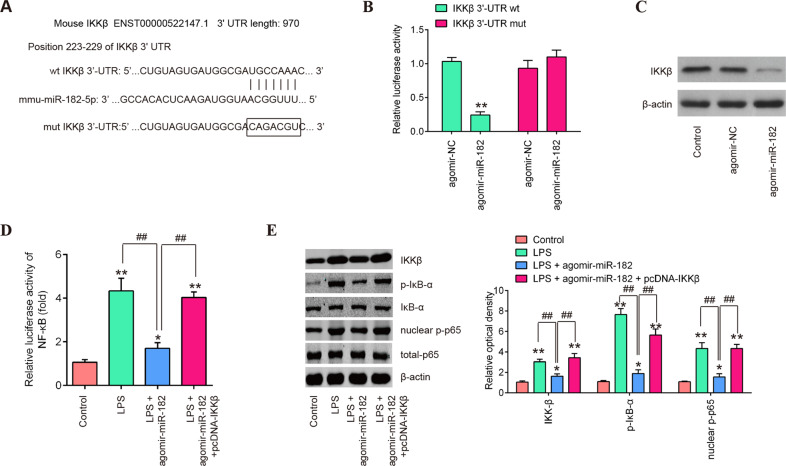
IKKβ is a direct target of miR-182 in BV-2 cells. **A** The predicted miR-182 binding sites on IKK*β*. **B** Luciferase assay of BV-2 cells (1 × 10^6^/well) co-transfected with firefly luciferase constructs containing the IKK*β* wild-type or mutated 3′-UTRs and agomir-miR-182 or agomir-NC, as indicated (*n* = 3). Data represent the mean ± SD of three independent experiments. **p < 0.01 vs. agomir-NC group. **C** The protein levels of IKK*β* were detected by western blot after agomir-miR-182 transfection. Agomir-miR-182 and pcDNA-IKKβ were co-transfected into the cultured BV-2 cells 4 h prior to LPS treatment and incubated for 24 h. **D** The NF-κB signaling pathway activity in BV-2 cells was measured by a Promega luciferase assay kit. **E** The protein expression levels of IKKβ, p-IκBα, IκBα, nuclear-p-p65 and total p65 were detected by western blot analysis. Data represent the mean ± SD of three independent experiments. *p < 0.05, **p < 0.01 vs. Control grou*p*; ^##^*p* < 0.01 vs. LPS + agomir-NC group.

To explore the underlying molecular mechanisms involved in the miR-182-mediated protective in LPS-induced apoptosis and inflammatory responses, we relied on TargetScan 7.0 and miRanda to predict the targets of miR-182 and identified IKKβ as a potential target of miR-182 (Fig. 5A). Next, a luciferase reporter assay was performed in BV-2 cells to determine whether IKKβ is a direct target of miR-182. As shown in Fig. 5B, agomir-miR-182 significantly repressed the luciferase activity of the IKKβ-3′UTR wt reporter plasmid. However, there is no change of luciferase activity in the cells co-transfected with aromir-miR-182 and the IKKβ-3′UTR mut reporter plasmid. Furthermore, miR-182 overexpression decreased protein level of IKKβ in BV-2 cells, as determined by western blot analysis (Fig. 5C), suggesting that miR-182 suppressed the translation of IKKβ in BV-2 cells.

Since IKKβ is a significant activator of the NF-κB pathway, which is closely associated with the inflammatory response in SCI [19, 44]. To examine whether miR-182 influences the activation of the NF-κB signaling pathway, we examined the activation of the NF-κB signaling pathway in LPS-treated BV-2 cells by using a reporter assay. Luciferase activity was significantly increased in LPS group, compared to control group. However, when agomir-miR-182 was transfected into LPS-treated cells, there was a significant decrease in luciferase activity in this cell culture model. Importantly, the decreased luciferase activity caused by agomir-miR-182 was reversed by overexpression of IKKβ in the cell culture model (Fig. 5D). Besides, western blot results showed that LPS treatment led to a significant increase in the protein expression of NF-κB pathway-related proteins, IKKβ, p-IκBα and nuclear-p-p65, compared to the control group, indicating that LPS activated the NF-κB pathway in BV-2 cells. In contrast, agomir-miR-182 transfection attenuated the promoting effect of LPS on these proteins. However, the downregulated protein levels of p-IκBα, nuclear-p-p65 and IKKβ induced by miR-182 were reversed when IKKβ was overexpressed (Fig. 5E). All these results indicated that the effect of miR-182 on the NF-κB pathway activation is dependent on the targeting of IKKβ.

### miR-182 protects BV-2 cells from LPS-induced apoptosis and the inflammatory response by targeting IKKβ

**Fig. 6:**
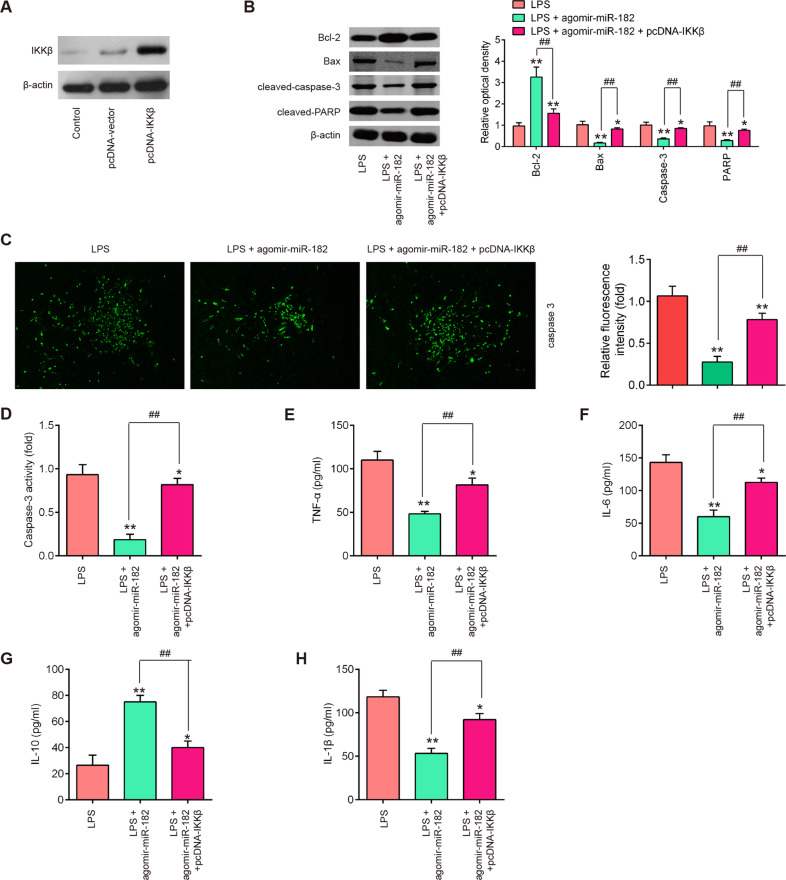
miR-182 protects BV-2 cells from LPS-induced apoptosis and the inflammatory response by targeting IKKβ. Agomir-miR-182 and pcDNA-KKβ were co-transfected into the cultured BV-2 cells (1 × 10^6^/well) 4 h prior to LPS treatment, and incubated for 24 h, then cells were harvested for next experiments. **A** The transfected efficiency of of IKKβ was determined by western blot. **B** The protein expression levels of Bcl-2, Bax, cleaved-caspase-3 and cleaved-PARP were detected by western blot analysis. **C** The protein expression level of caspase-3 was detected by IFA. **D**, Activity of caspase-3 was measured using a commercial kit. **E**–**H**, The expression of TNF-α, IL-6, IL-10, and IL-1β were measured by ELISA analysis. Data represent the mean ± SD of three independent experiments. *p < 0.05, **p < 0.01 vs. Control group; ^##^*p* < 0.01 vs. LPS + agomir-miR-182 group.

IKKβ was a target of miR-182 in BV-2 cells, therefore, we sought to test whether IKKβ mediated the protection of miR-182 on LPS-induced apoptosis and inflammatory responses. The IKKβ expression vector, pcDNA-IKKβ and agomir-miR-182 were co-transfected into BV-2 cells 4 h prior to LPS treatment. As shown in Fig. 6A, the protein expression of IKKβ was notably increased after pcDNA-IKKβ transfection in BV-2 cells. Functionally, compared with LPS group, agomir-miR-182 decreased Bax, cleaved caspase-3 and cleaved-PARP protein expression level and increased the Bcl-2 expression, and the effects were reversed by overexpression of IKKβ in BV-2 cells (Fig. 6B). IFA results showed that agomir-miR-182 suppressed LPS-induced caspase-3 expression, and the effect was also reversed by overexpression of IKKβ (Fig. 6C). Similar results were observed in the activity of caspase-3 (Fig. 6D). Besides, ELISA was performed to evaluate inflammatory cytokine productions in LPS-treated BV-2 cells following pcDNA-IKKβ and agomir-miR-182 co-transfection. As shown in Fig. 6E–H, agomir-miR-182 treatment resulted in the reduction of TNF-α, IL-6 and IL-1β productions and the induction of the IL-10 production in LPS-treated BV-2 cells, whereas the anti-inflammatory effects of miR-182 were reversed by overexpression of IKKβ. These data suggest that miR-182 protects BV-2 cells from LPS-induced apoptosis and inflammatory responses by targeting IKKβ.

### Agomir-miR-182 blocked activation of the NF-κB pathway in vivo

**Fig. 7:**
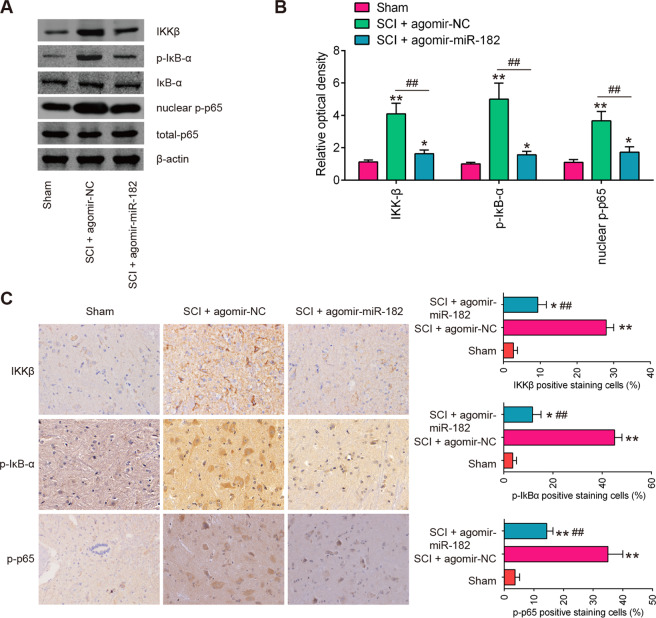
miR-182 blocked the activation of the NF-κB pathway in vivo. The mice were subjected to SCI and treated intrathecally with agomir-miR-182/agomir-NC (2 μl, 20 µM). At 7 days post injury, the animals were sacrificed following pentobarbital sodium (50 mg/kg, i.p) anesthesia, and subsequently, the spinal cord tissues were harvested for further experiments. **A** The protein expression levels of IKKβ, p-IκBα, IκBα, nuclear-p-p65, and total p65 were detected by western blot analysis (*n* = 6/group). **B** The bands were semi-quantitatively analyzed by using Image J software, normalized to β-actin density. **C** The expression of IKKβ, p-IκBα, and nuclear-p-p65 was detected by IHC. Data represent the mean ± SD of three independent experiments. *p < 0.05, **p < 0.01 vs. Sham group; ^##^*p* < 0.01 vs. SCI + agomir-NC group.

To test whether miR-182 influences the NF-κB pathway activation in vivo, western blot was performed to analyze the expression levels of NF-κB pathway-related core factors in mice following SCI. As shown in Fig. 7A, B, the expression levels of IKKβ, p-IκB-α and nuclear p-p65 were markedly upregulated in SCI mice compared with sham group, indicating that LPS activates the NF-κB pathway. However, the activation of the NF-κB pathway was blocked when agomir-miR-182 was treated, as evidenced by the reduction of IKKβ, p-IκB-α and nuclear p-p65 expression. We also measured the expression of IKKβ, p-IκB-α and nuclear p-p65 in in spinal cord tissues of SCI mice using IHC. Compared with the sham group, the expression of IKKβ, p-IκB-α and nuclear p-p65 protein in the SCI group was significantly increased. After treatment with agomir-miR-182, the protein expression levels of IKKβ, p-IκB-α and nuclear p-p65 were downregulated to varying degrees relative to the SCI group (Fig. 7C). These data suggest that miR-182 blocked NF-κB pathway activation by suppressing IKKβ expression in vivo.

## Discussion

**Fig. 8:**
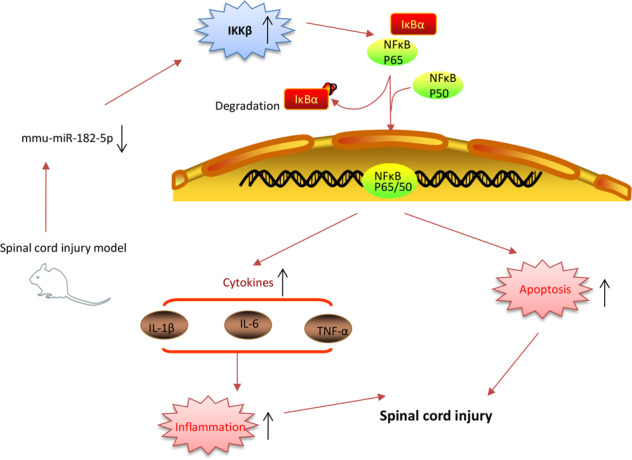
A schematic illustration of the proposed model depicting miR-182 improves spinal cord injury in mice by modulating apoptosis and the inflammatory response via IKKβ/NF-κB. Schematic diagrams showing that miR-182 was downregulated in SCI mice, and induced the expression of its target IKKβ, thus promoting the activation of the NF-κB pathway, resulting in an inflammatory response and apoptosis, leading to secondary damage.

In the present study, miR-182 was significantly downregulated in spinal cord tissues of SCI mice. Overexpression of miR-182 improved the functional recovery of SCI mice through suppression of apoptosis and the inflammatory response. Using an LPS-induced BV-2 cell injury model, we found that overexpression of miR-182 protected BV-2 cells against LPS-induced apoptosis and the inflammatory response by suppressing the IKKβ/NF-κB pathway. Notably, our data indicate that the overexpression of miR-182 exerted protective effects on the inflammatory response by blocking IKKβ/NF-κB pathway activation in vivo and in vitro (Fig. 8). These findings suggest that miR-182 may be a potential target for the treatment of SCI.

A number of studies have demonstrated that miRNAs are aberrantly expressed in spinal cord tissues and associated with the SCI-induced inflammatory response and apoptosis [16, 45, 46]. For example, Zhou et al. found that miR-34a was downregulated in spinal cord tissues from SCI rat model, and miR-34a upregulation alleviated SCI through reducing apoptosis and inflammation via inhibition of high mobility group box-1 expression in TLR4 signaling [47]. Chen et al. showed that miR-92b-3p promoted functional recovery of SCI mice through the PTEN/AKT pathway [48]. Dai et al. found that miR-210 was significantly reduced in SCI mice, and miR-210 overexpression alleviated the progression of SCI by inhibiting inflammation [49]. In the present study, we analyzed GSE19890 from the GEO database to determine the expression pattern of miRNAs in spinal cord tissues of SCI mice. According to the GEO database, we found that several miRNAs including miR-182, miR-21, miR-223 miR-124 and miR-126, were abnormal in the spinal cord tissues, which are consistent with previous reports [20, 24, 34, 35], indicating the reliability of this microarray. Among these miRNAs, we found miR-182 was the most downregulated miRNA in spinal cord tissues from SCI mice, suggesting the involvement of miR-182 in SCI.

The function of miR-182 in inflammation has been extensively investigated. For example, Jiang et al. found that miR-182 alleviated liver injury through inhibiting TLR4-mediated inflammatory reaction in a hepatic ischemia-reperfusion (I/R) injury rat model [39]. Wang et al. has demonstrated that miR-182 improved the cerebral injury via suppressing the TLR4-mediated inflammation [36]. Notably, a recent study showed the neuroprotective effect of miR-182 in mice following SCI by suppressing the neurons apoptosis [41]. These findings prompted us to investigate the role of miR-182 in inflammatory responses in SCI. Here we conducted a miR-182-based strategy to treat SCI using the agomir-miR-182. We found that agomir-miR-182 injection could improve the functional recovery, reduce lesion size and spinal cord water edema after SCI. Given the destructive role of the inflammatory response and apoptosis in SCI progression, we also inferred that miR-182 might affect the neuronal inflammatory response and apoptosis in SCI. As expected, forced expression of miR-182 effectively suppressed the inflammatory response and apoptosis in SCI mice. Consistent with the in vivo results, it was also observed that miR-182 overexpression exhibited its protective effects against the LPS-induced inflammatory response and apoptosis using LPS-induced BV-2 cell injury model. The data suggest that miR-182 protects the spinal cord from secondary damage and facilitate recovery after SCI in mice.

IKKβ, one component of the IKK complex, has received attention in SCI for its essential contributions to IκB phosphorylation and NF-κB activation [7]. For example, Kang et al. found that IKKβ-mediated neutrophil activation in the injured spinal cord exacerbated inflammation and neuronal damage and impeded functional recovery after SCI [50]. Several studies have shown that the activation of IKKβ in SCI is finely regulated by miRNAs. Deng et al. reported that miR-136-5p ameliorated the SCI through repressing the inflammatory cell infiltration by directly targeting IKKβ [51]. Zhou et al. demonstrated that miR-199b attenuated acute SCI through regulation of the IKKβ/NF-κB signaling pathway [29]. However, whether IKKβ is involved in the protection of miR-182 against SCI remains unclear. In our study, IKKβ was confirmed as a direct target of miR-182 in SCI cell culture model. In addition, the protective effects of miR-182 against LPS-induced apoptosis and inflammatory response were abrogated by the overexpression of IKKβ, suggesting that miR-182 protected against LPS-induced BV-2 cell injury via suppressing the expression of IKKβ.

It is well known that the IKKβ is an inducer of the NF-κB pathway through promotion of phosphorylation of the IκB protein [10, 52, 53]. Activation of the NF-κB signaling pathway has been closely associated with the inflammatory response after SCI [54]. Previous studies have reported that miRNAs play key roles in inflammatory response through the suppression of the NF-κB pathway. Yang et al. showed that miR-146a could suppress inflammatory response in SCI through inhibition of NF-κB pathway [30]. Given IKKβ is directly targeted by miR-182, we hypothesized that miR-182 suppress the inflammatory responses by the IKKβ/NF-κB pathway. In this study, it was observed that the miR-182 overexpression significantly inhibited the activation of NF-κB in vitro and in vivo, which can be used to partially explain the reduction of inflammatory cytokines. Furthermore, overexpression of IKKβ reactivated the NF-κB pathway blocked by miR-182 in SCI cell culture model. These results suggest that miR-182 exerts its protective effects against SCI-induced inflammatory response by inhibiting the IKKβ/NF-κB signaling pathway.

Many studies have reported the protective effects of miRNAs from different cellular source types including neuron and astrocytes, during the progression of SCI. For example, Zhang et al. found that miR-21 was upregulated in neurons after SCI, and overexpression of miR-21 reduced neuronal apoptosis by targeting PDCD [55]. Lv et al. showed that overexpression of miR-448 improved spinal motor neuron regeneration by regulating the PI3K/AKT/ Bcl-2 axis [56]. Yuan et al. reported that miR-124 inhibited primary spinal neuronal apoptosis by suppressing the GCH1 expression [57]. In the central nervous system, astrocytes are the most abundant and widely distributed glial cells and it has been considered as the ideal therapeutic target cells for SCI [58, 59]. For example, Bhalala et al. found miR-21 was highly expressed in astrocytes in injured spinal cord and inhibition of miR-21 attenuated astrocytic hypertrophy and glial scar progression following SCI [60]. In addition, in microglia, miR-223-5p was upregulated in M1 microglia, and miR-223-5p inhibition suppressed the inflammatory response and reduced glia reaction and neuron apoptosis in SCI 34. In this study, we observed that the injury site of SCI mice with abundant glial cells proliferation and satellitosis, suggesting miR-182 may exert its protective effect through regulating glial cell biological functions. Thus, we chose a microglia cell line (BV2) for subsequent experiments. Further investigation showed that overexpression of miR-182 suppressed the LPS-induced inflammatory response and apoptosis in BV-2 cells, which is consistent the in vivo results. Therefore, we concluded that the cellular miR-182 from glial may play an important role in protection against SCI. However, whether other cellular miR-182 also participate in protection against SCI need further study.

In conclusion, we demonstrated that miR-182 improved secondary damage and facilitate recovery by inhibiting the inflammatory responses and apoptosis via inactivation of the IKKβ/NF-κB pathway (Fig. 8). Our findings suggest that miR-182 may be a therapeutic target of SCI.

## Data Availability

The data used to support the findings of this study are included within the article.
